# Normal imaging findings after ascending aorta prosthesis implantation on ^18^F-Fluorodeoxyglucose Positron Emission Tomography with computed tomography

**DOI:** 10.1007/s12350-021-02826-0

**Published:** 2021-10-27

**Authors:** Ali R. Wahadat, Wilco Tanis, Ties A. Mulders, Laura H. Graven, Margreet W. A. Bekker, Jos A. Bekkers, Jolien W. Roos-Hesselink, Ricardo P. J. Budde

**Affiliations:** 1 Department of Radiology and Nuclear Medicine, Erasmus Medical Center, ND-547, Dr. Molewaterplein 40, 3015 GD Rotterdam, The Netherlands; 2 Department of Cardiology, Thoraxcenter, Erasmus Medical Center, Rotterdam, The Netherlands; 3 Department of Cardiology, Haga Teaching Hospital, The Hague, The Netherlands; 4 Department of Cardiothoracic Surgery, Thoraxcenter, Erasmus Medical Center, Rotterdam, The Netherlands

## Abstract

**Background:**

To diagnose abnormal ^18^F-Fluorodeoxyglucose (^18^F-FDG) uptake in suspected endocarditis after aortic root and/or ascending aorta prosthesis (ARAP) implantation, it is important to first establish the normal periprosthetic uptake on positron emission tomography with computed tomography (PET/CT).

**Methods:**

Patients with uncomplicated ARAP implantation were prospectively included and underwent ^18^F-FDG-PET/CT at either 12 (± 2) weeks (group 1) or 52 (± 8) weeks (group 2) after procedure. Uptake on three different locations of the prosthesis (“cranial anastomosis (CA),” “prosthetic heart valve (PHV),” “ascending aorta prosthesis (AAP)”) was scored visually (none/low/intermediate/high) and quantitatively (maximum standardized uptake value (SUV_max_) and target-to-background ratio (SUV_ratio_).

**Results:**

In total, 20 patients (group 1: n = 10, group 2: n = 10) (mean age 64±7 years, 70% male) were included. Both groups had similar visual uptake intensity for all measured areas (CA: mostly low-intermediate (16/20 (80%)), *p* = .17; PHV: low-intermediate (16/20 (80%)), *p* = .88; AAP: low-intermediate (19/20 (95%)), *p* = .48). SUV_max_ for CA was 5.6 [4.1-6.1] and 3.8 [3.1-5.9] (median [IQR],* p* = .19), and around PHV 5.0 [4.1-5.7] and 6.3 [4.6-7.1] (*p* = .11) for groups 1 and 2, respectively. SUV_ratio_ for CA was 2.8 [2.3-3.2] and 2.0 [1.7-2.6] (median [IQR], *p* = .07) and around PHV 2.5 [2.4-2.8] and 2.9 [2.3-3.5] (median [IQR], *p* = .26) for groups 1 and 2, respectively.

**Conclusion:**

No significant differences were observed between PET/CT findings at 3 months and 1 year after ARAP implantation, warranting caution in interpretation of PET/CT in the first year after implantation.

**Supplementary Information:**

The online version contains supplementary material available at 10.1007/s12350-021-02826-0.

## Introduction

Infective endocarditis (IE) and especially prosthetic valve endocarditis (PVE) are difficult to diagnose.^1,2^ The diagnosis becomes even more challenging when an implanted aortic prosthetic heart valve is combined with an ascending aorta and root conduit (Bentall graft) or a supracoronary ascending aorta prosthetic replacement (SCAR), since there are no specific criteria for the diagnosis of infection of these prostheses.^3^ Normal imaging findings on computed tomography angiography (CTA) after a recent Bentall procedure often show periaortic fluid to be present around the prosthesis.^4,5^ ^18^F-Fluorodeoxyglucose (^18^F-FDG) positron emission tomography with computed tomography (PET/CT) is nowadays used as an additional diagnostic tool for the diagnosis of PVE according to the latest European Society of Cardiology (ESC) guidelines for IE.^1^ However, these guidelines do not mention the use of ^18^F-FDG PET/CT for the detection of aortic root and/or ascending aorta prosthesis (ARAP) infection, while this technique is increasingly used. Recently, two prospective studies described the normal ^18^F-FDG uptake patterns and intensities around prosthetic heart valves (PHVs) and showed no significant differences between ^18^F-FDG uptake around prosthetic valves at different time points within the first year after implantation.^6,7^ It is generally assumed that normal healing response after replacement of the ascending aorta and root will result in ^18^F-FDG uptake at the operated area similar to what is seen in prosthetic valve implantation. It is not well known how long this process will take and how long the PET-CT may be relatively unreliable. However, the normal ^18^F-FDG uptake intensity and pattern on ARAP needs to be known, to enhance correct interpretation of ^18^F-FDG PET/CT scans in patients with suspected infection. In order to determine the normal ^18^F-FDG uptake patterns and intensity around ARAP, we prospectively assessed the visual and quantitative ^18^F-FDG uptake at two different time points and three different locations in the aorta in the first year after Bentall and SCAR procedures.

## Materials and Methods

### Patient Selection and Classification

In this prospective cross-sectional study, patients 50 years or older who had undergone an uncomplicated Bentall or SCAR procedure were included. An uncomplicated procedure was defined as a procedure with no complications during or directly after surgery. A detailed list of the inclusion and exclusion criteria is presented in Table 1. The medical ethics committee approved the study (NL42743.041.12). All patients provided written informed consent. Patients were included and underwent PET-CT after the Bentall/SCAR procedure at either 12 (± 2) weeks (group 1) or 52 (± 8) weeks (group 2).

**Table 1:** Inclusion and exclusion criteria

Inclusion criteria	Exclusion criteria
Age ≥ 50 years Patients after uncomplicated Bentall/SCAR procedure in aortic position including a PHV Normal routine follow-up TTE (standardly performed 5 days after surgery) or intra-operative TEE. With no signs of obstruction, endocarditis or significant paravalvular leakages. Weight < 110 kg	Diabetes mellitus Mild contractile dysfunction of the left and/or right ventricle (eyeballing, Ejection fraction < 45%, TAPSE < 14 mm) Active cardiac decompensation Uncontrolled cardiac arrhythmias Suspicion of active endocarditis Previous participation in scientific studies using radiation. (Possible) pregnancy in pre-menopausal women above 50 years not on reliable birth control therapy. Use of pericardial patches and re-operation of aortic PHV in past medical history Refusal to be informed about potential FDG-PET findings

^*SCAR*, supracoronary ascending aorta replacement; *PHV*, prosthetic heart valve; *TTE*, transthoracic echocardiogram; *TEE*, transesophageal echocardiogram; *TAPSE*, tricuspid annular plane systolic excursion; *FDG-PET*, fluorodeoxyglucose-Positron Emission Tomography^

Included patients did not have any clinical signs of IE or other infection (fever, shivers, dyspnea, etc.) at the time of the ^18^F-FDG PET/CT.

### Image Acquisition

#### ^18^F-FDG PET/CT

To induce free fatty acid metabolism and suppress myocardial glucose metabolism, patients followed a 12 hours low-carbohydrate diet followed by 12 hours fasting.^8-10^ Thereafter, patients received an intravenous ^18^F-FDG-injection of 2.0 MBq/kg. Patients were hydrated with 1000 mL of water 1 hour prior to image acquisition. Blood glucose levels were checked before ^18^F-FDG injection and the limit was set to 8.9 mmol·L^−1^. Approximately 1 hour after ^18^F-FDG injection, the PET/CT was performed using a Biography Sensation 16scanner (SIEMENS Medical, Germany). Before the PET acquisition, a low-dose CT scan was performed for attenuation correction. A PET-scan of the heart was then obtained with 3-minute acquisitions per bed position using a 3-dimensional acquisition mode. Attenuation-corrected PET images were reconstructed with an ordered-subset expectation-maximization iterative reconstruction algorithm.

### Image Analysis and Interpretation

#### ^18^F-FDG PET/CT Analysis

**Figure 1:**
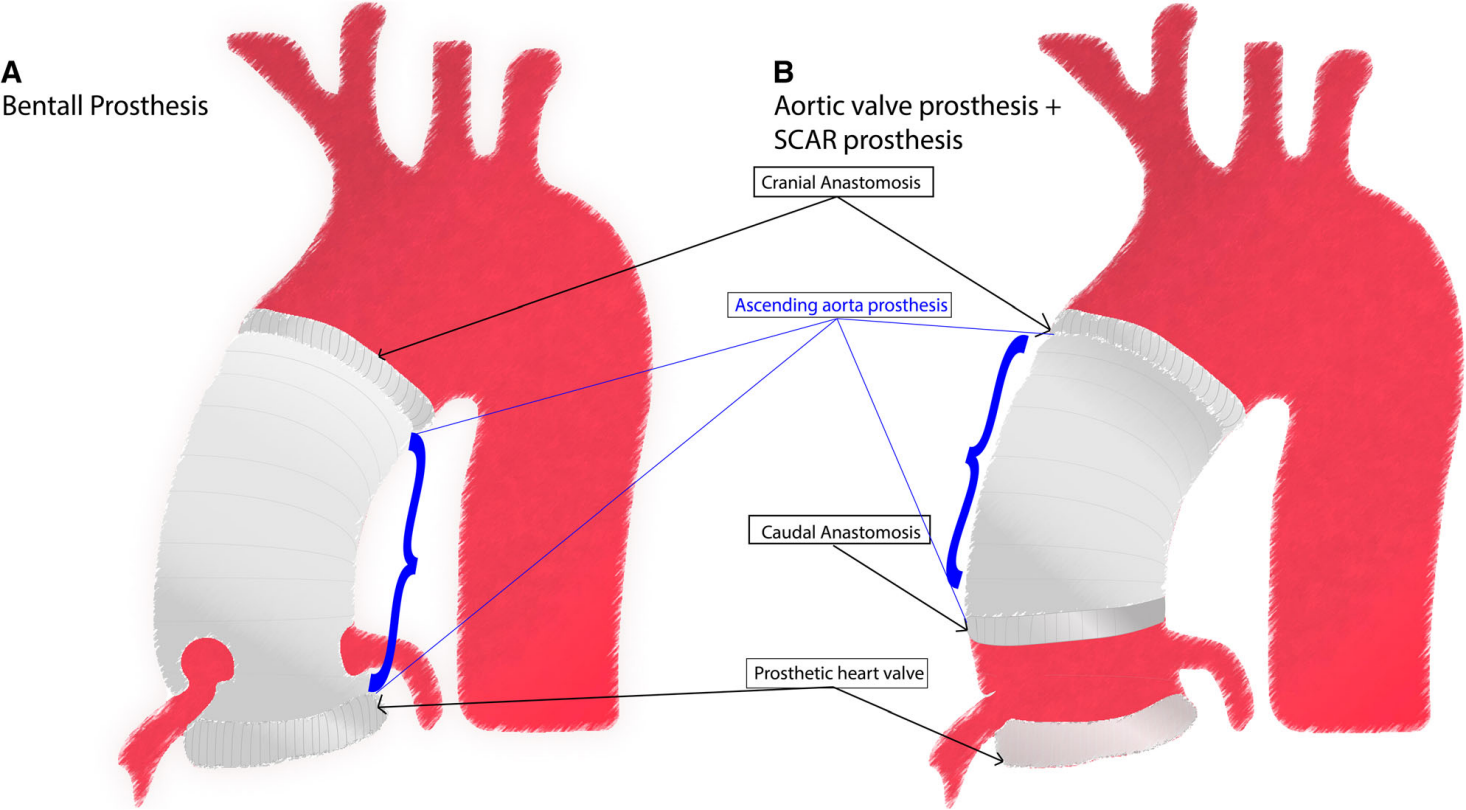
Schematic view of the Bentall prosthesis (**A**) and SCAR prosthesis + aortic prosthetic heart valve (**B**) and the areas of measured FDG activity indicated by arrows

Uptake of ^18^F-FDG on three different levels (cranial anastomosis, around the PHV, and on the ascending aorta prosthesis) were scored visually and if feasible also quantitatively for all patients by experienced nuclear medicine physicians (TM, LG) who were blinded for group allocation (Figure 1). Additional visual and quantitative measurements were also made at the caudal anastomosis for patients with a SCAR procedure including a PHV implantation. For patients with a Bentall procedure, the caudal anastomosis equalled the area of the PHV. The measured area of “the ascending aorta prosthesis” was defined as the part of the prosthesis between the cranial and caudal anastomosis. Figure 1 illustrates the measured levels on both prostheses. For qualitative analyses, the qualification visual score for hypermetabolism (QVSH) was used, scoring the uptake as “none” (no or less than blood pool uptake), “low” (more than blood pool uptake but less than in the liver), “intermediate” (more than liver uptake), or “high” (intense uptake). “Blood pool” uptake was defined as the mean uptake in the blood pool of the descending aorta at the level of the left atrium. Distribution patterns were scored as either “focal” (solitary ^18^F-FDG uptake spot) or “multi focal” (> 1 solitary ^18^F-FDG uptake spot) versus “diffuse” (> 1 location of ^18^F-FDG uptake that cannot be differentiated as solitary spots) which could be homogeneous (overall same level of ^18^F-FDG uptake intensity) or heterogeneous (different levels of ^18^F-FDG uptake intensity). Quantitative analyses were performed by measuring the maximum standardized uptake value (SUV_max_) and target-to-background ratio (SUV_ratio_) on standardized European Association of Nuclear Medicine Research Ltd. (EARL) and non-EARL reconstructions using commercially available software (Carestream v12.2.2.1025). SUV_max_ was measured in an automated volume of interest (VOI) around both anastomoses, which was visually verified to include the whole anastomotic area. The SUV_ratio_ was then calculated as the ratio of the SUV_max_ and the mean SUV in the blood pool of the descending aorta, taking care not to include the vessel wall.

Myocardial suppression was scored as “fully suppressed” (no uptake), “low” (more than mediastinal uptake but less than in the liver), “intermediate” (more than liver uptake), “high focal” (much more than liver uptake, but focal), “high diffuse” (much more than liver uptake, diffuse).

Statistics

Descriptive statistics were used for analysis of the outcomes. For continuous variables, means and standard deviations (SD) were used in case of normal distribution. In case of non-normal distribution, medians and interquartile ranges (IQR) were used. The IQR and confidence interval (CI) were denoted in square brackets. Comparisons between groups were made using the Chi-square test for categorical variables and non-parametric test (Mann–Whitney U) for continuous variables. A significance level of *p* = .05 and 95% confidence intervals (CI) were used.

## Results

### Patients Characteristics and Classification

A total of 20 patients were included in either group 1 (n = 10) or group 2 (n = 10). Age was (median with IQR) 64 [60-71] years, (group 1 = 64 [60-70]; group 2 = 62 [59-73]) and most of the patients were male (n = 14, 70%). A Bentall procedure was performed in 14/20 (70%) patients (group 1 = 6, group 2 = 8) and SCAR in 6/20 (30%) patients (group 1 = 4, group 2 = 2). All patients with SCAR also underwent a concomitant aortic valve replacement (AVR). There were 9 (45%) biological and 11 (55%) mechanical prosthetic valves, not significantly different between groups (*p* = .18). Surgical adhesives such as BioGlue that are known to be FDG-avid were not used during any of the implantations. No patient was suspected of having endocarditis at the time of operation or the PET/CT scan. Baseline characteristics of the participants are summarized in Table 2.

**Table 2:** Baseline characteristics of all patients and of patients in groups 1 and 2

	All included patients	Group 1 (12 (± 2) weeks after prosthesis implantation)	Group 2 (12 (± 2) months after prosthesis implantation)	*p* value***
Number of patients	20	10	10	
Age, median [IQR], years	64[60-71]	64[60-70]	62[59-73]	.38
Gender, n(%)				1
Male	14(70)	7(70)	7(70)	
Female	6(30)	3(30)	3(30)	
BMI, median [IQR], kg·m^−2^	26[23-29]	24[23-28]	28[24-31]	.12
Days between surgery and PET/CT, median [IQR], days	218[90-374]	91[88-95]	373[358-414]	<.01
Laboratory results*				
Serum levels of leucocytes × 10^9^/L, median [IQR]	10.8 [8.9-12.9]	10.6 [9.8-13.6]	10.8 [8.5-12.8]	.58
Serum levels of creatinine µmol·L^−1^, median [IQR]	82 [62-91]	77 [58-105]	86 [67-90]	.8
Medical history, n(%)				
Hypertension	2 (10)	1 (10)	1 (10)	1
Atrial fibrillation	4 (20)	2 (20)	2 (20)	1
Heart failure	0 (0)	0 (0)	0 (0)	NA
Myocardial infarction	1 (5)	1 (10)	0 (0)	.31
Prior thoracic surgery	0 (0)	0 (0)	0 (0)	NA
Procedure				.33
Bentall	14 (70)	6 (60)	8 (80)	
AVR+SCAR	6 (30)	4 (40)	2 (20)	
PHV type, n(%)				
Mechanical	11 (55)	4 (40)	7 (70)	.18
Biological	9 (45)	6 (60)	3 (30)	
Valve manufacturer, n(%)				
St. Jude	11 (55)	4 (40)	7 (70)	.18
Perimount	9 (45)	6 (60)	3 (30)	
Valve size median [IQR] (mm)	26 [23-27]	26 [23-27]	26 [23-28]	.91
Aorta prosthetic size median [IQR] (mm)	28 [26-30]	29 [27-30]	28 [26-29]	.22
Surgery, n(%)				
Concomitant CABG	17 (85)	8 (80)	9 (90)	.53
Other concomitant procedure**	9 (45)	6 (60)	3 (30)	.37
Use of surgical adhesives	0 (0)	0 (0)	0 (0)	NA

*PHV*, prosthetic heart valve; *AVR*, aortic valve replacement; *BMI*, Body Mass Index; *PET/CT*, Positron Emission Tomography with computed tomography; *SCAR*, supracoronary aortic replacement; *CABG*, coronary artery bypass graft; *IQR*, interquartile range; *SD*, standard deviation

*Serum Leucocytes and Creatinine levels were measured as part of clinical practice ±5 days after surgery

**Nine patients underwent a concomitant procedure with the aortic PHV implantation containing two patients with a left atrial appendage amputation and pulmonary vein isolation procedure, seven patients with a hemiarch replacement

***Statistical difference between the two groups 1 and 2

### ^18^F-FDG PET/CT Findings

The median time between the surgery and ^18^F-FDG PET/CT was 91 [88-95] and 373 [358-414] days for groups 1 and 2, respectively (*p* < .01). Mean ± SD ^18^F-FDG dosage was 164 ± 30 MBq and not significantly different between the groups (*p* = .08). Preparation according to carbohydrate diet protocol was followed by all patients. In Table 3, a detailed presentation of the myocardial suppression as well as visual analysis of the ARAP in all patients is provided.

**Table 3:** Visual ^18^F-FDG PET/CT findings for all patients and for each patient per group

	All included patients	Group 1 (12 (± 2) weeks after prosthesis implantation)	Group 2 (12 (± 2) months after prosthesis implantation)	*p* value*
Number of patients	20	10	10	
FDG dose, MBq/kg, (mean±SD)	164 ± 30	152 ± 20	176 ± 34	.08
Time between FDG dose and start scan (min), m[IQR]	58 [57–62]	58 [57–63]	59 [57–62]	.77
Serum levels of glucose mmol·L (mean±SD)	5.6 ± .6	5.8 ± .5	5.5 ± .7	.23
Preparation according to carbohydrate diet protocol, n(%)	20 (100)	10 (100)	10 (100)	1
Myocardial suppression, n(%)				.33
Fully suppressed	11 (55)	5 (50)	6 (60)	
Low uptake	2 (10)	2 (10)	0 (0)	
Intermediate uptake	3 (15)	1 (10)	2 (20)	
High focal uptake	0 (0)	0 (0)	0 (0)	
High diffuse uptake	4 (20)	2 (20)	2 (20)	
Visual score cranial anastomosis (QVSH), n(%)				.17
None	2 (10)	0 (0)	2 (20)	
Low	10 (50)	4 (40)	6 (60)	
Intermediate	6 (30)	4 (40)	2 (20)	
High	2 (10)	2 (20)	0 (0)	
Specific FDG uptake pattern, n(%)				.94
Focal	7 (35)	4 (40)	3 (30)	
Multifocal	0 (0)	0 (0)	0 (0)	
Diffuse homogeneous	5 (25)	3 (30)	2 (20)	
Diffuse heterogeneous	6 (30)	3 (30)	3 (30)	
Visual score PHV (QVSH), n(%)				.88
None	0 (0)	0 (0)	0 (0)	
Low	9 (45)	5 (50)	4 (40)	
Intermediate	7 (35)	3 (30)	4 (40)	
High	4 (20)	2 (20)	2 (20)	
Specific FDG uptake pattern, n(%)				.31
Focal	1 (5)	1 (10)	0 (0)	
Multifocal	0 (0)	0 (0)	0 (0)	
Diffuse homogeneous	19 (95)	9 (90)	10 (100)	
Diffuse heterogeneous	0 (0)	0 (0)	0 (0)	
Visual score total prosthesis (QVSH), n(%)				.48
None	1 (5)	1 (10)	0 (0)	
Low	12 (60)	5 (50)	7 (70)	
Intermediate	7 (35)	4 (40)	3 (30)	
High	0 (0)	0 (0)	0 (0)	1
Specific pattern FDG uptake, n(%)				
Focal	0 (0)	0 (0)	0 (0)	
Multifocal	0 (0)	0 (0)	0 (0)	
Diffuse homogeneous	18 (90)	9 (90)	9 (90)	
Diffuse heterogeneous	2 (10)	1 (10)	1 (10)	

*Statistical difference between groups 1 and 2; *QVSH*, Qualification Visual Score of Hypermetabolism

**Figure 2:**
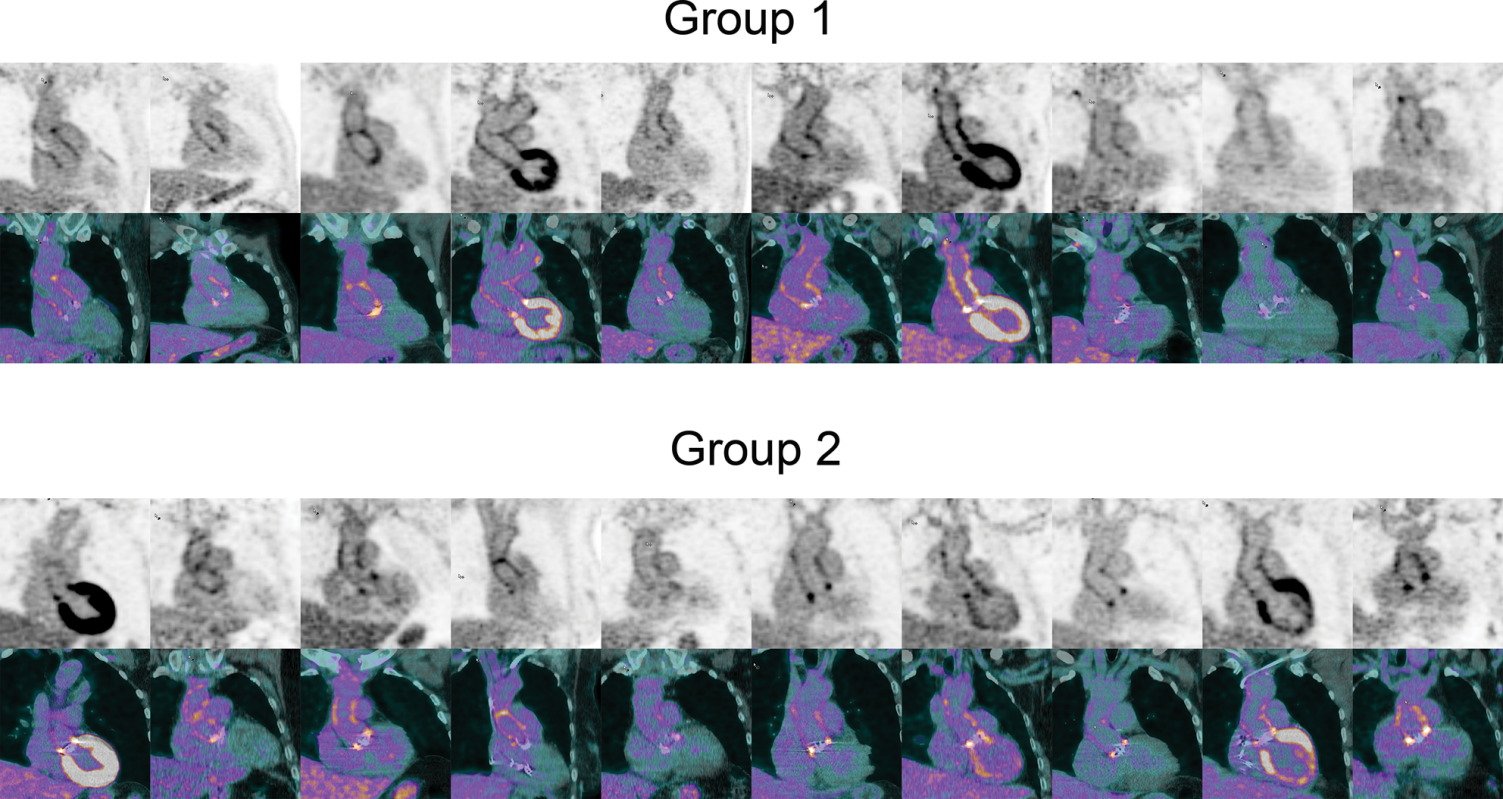
^18^F-FDG uptake around the prosthesis on coronal views of attenuation-corrected (AC) images and fused attenuation-corrected images with CT in all patients. Scaling was set the same for all AC images and represents SUV with a range of 0-7

**Figure 3:**
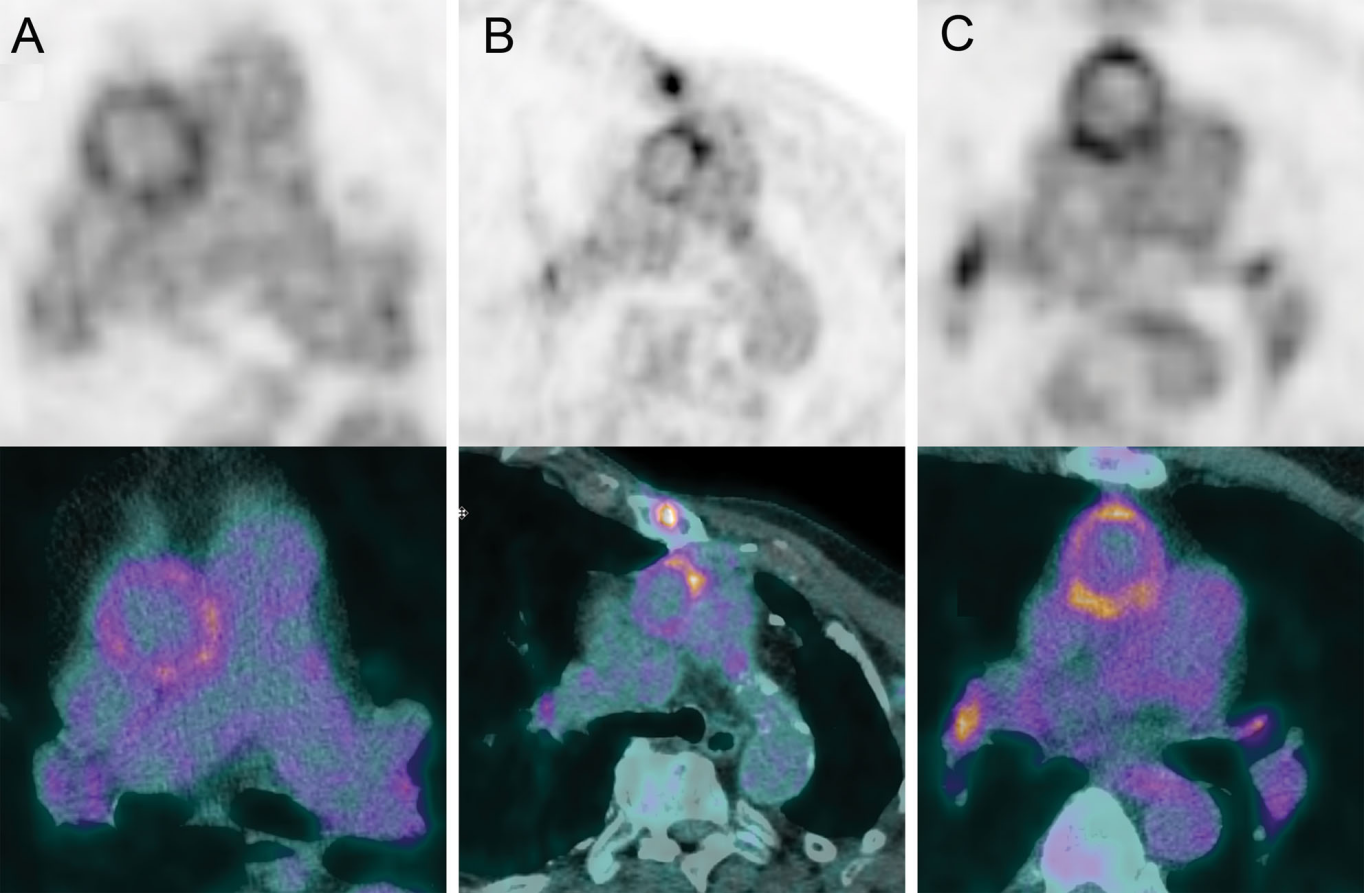
Examples of ^18^F-FDG uptake patterns (**A**: diffuse homogeneous, **B**: focal, **C**: diffuse heterogeneous) around the cranial anastomosis. Scaling was set the same for all images and represents SUV with a range of 0-7

**Figure 4:**
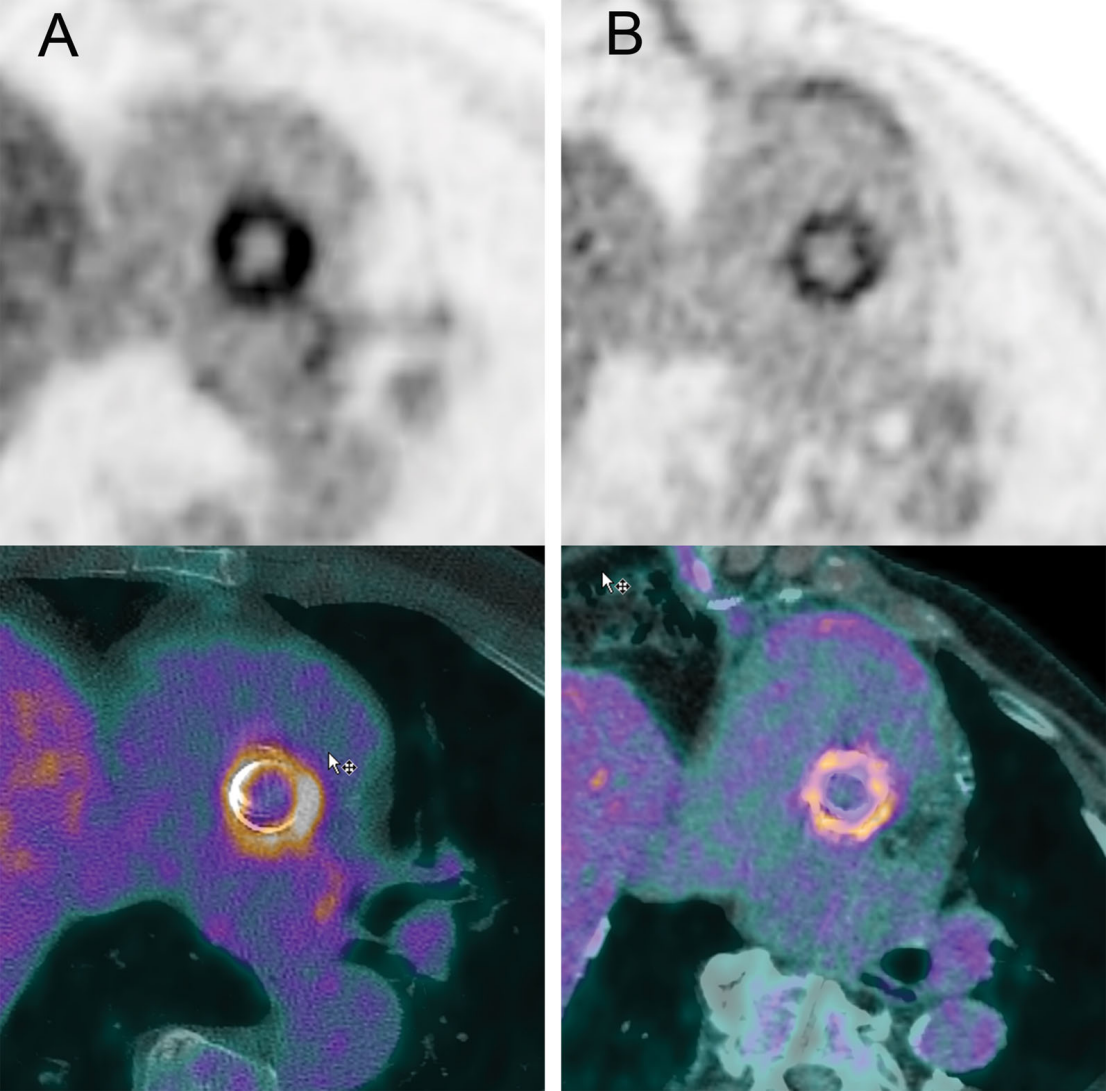
Examples of the diffuse homogeneous ^18^F-FDG uptake pattern around the prosthetic heart valve on a patient from group 1 (**A**) and a patient from group 2 (**B**). Scaling was set the same for all images and represents SUV with a range of 0-7

Figure 2 presents an overview of FDG activity around the prosthesis in all 20 patients included in this study. The QVSH around the three measured levels (cranial anastomosis, at the PHV, and at the ascending aorta prosthesis) showed no significant difference between the 2 groups (*p* = .17; *p* = .88; and *p* = .48, respectively) and was scored primarily as low or intermediate (16/20 (80%), 16/20 (80%), and 19/20 (95%), respectively). Details of the QVSH and the distribution patterns are provided in Table 3. Examples of the uptake patterns are demonstrated in Figure 3 with an example of diffuse homogeneous pattern in group 1 versus group 2 in Figure 4.

In Table 4, a detailed presentation of the quantitative analysis of ^18^F-FDG uptake is provided.

**Table 4:** Quantitative ^18^F-FDG PET/CT findings for all patients and for each patient per group

	All included patients	Group 1 (12 (±2) weeks after prosthesis implantation)	Group 2 (12 (±2) months after prosthesis implantation)	*p* value*
Number of patients	20	10	10	
SUV_max_ cranial anastomosis (mean±SD)	4.7 [3.3–6.1]	5.6 [4.1–6.1]	3.8 [3.1–5.9]	.19
SUV_ratio_ cranial anastomosis (mean±SD)	2.3 [1.9–3.0]	2.8 [2.3–3.2]	2.0 [1.7–2.6]	.07
EARL SUV_max_ cranial anastomosis (mean±SD)	3.4 [2.8–4.1]	3.8 [3.4–4.2]	3.1 [2.5–3.6]	.08
EARL SUV_ratio_ cranial anastomosis (mean±SD)	1.7 [1.4–1.9]	1.8 [1.7–2.0]	1.5 [1.3–1.8]	.04
SUV_max_ PHV median [IQR]	5.5 [4.2–6.8]	5.0 [4.1–5.7]	6.3 [4.6–7.1]	.11
SUV_ratio_ PHV median [IQR]	2.6 [2.4–3.3]	2.5 [2.4–2.8]	2.9 [2.3–3.5]	.26
EARL SUV_max_ PHV median [IQR]	4.2 [3.4–5.0]	4.1 [3.2–4.6]	4.5 [3.7–5.6]	.21
EARL SUV_ratio_ PHV median [IQR]	2.1 [1.7–2.5]	1.9 [1.7–2.2]	2.3 [1.8–2.6]	.21

*PHV*, prosthetic heart valve; *MBq/kg*, Megabecquerel/kilograms; *SUVmax*, maximum standardized uptake value; *SUVratio*, standardized uptake value ratio (target-to-background ratio); *EARL*, European Association of nuclear medicine Research Ltd.

*Statistical difference between groups 1 and 2

With the exception of SUV_ratio_ of the cranial anastomosis on the EARL reconstructed images, no significant difference was found in the quantitative analysis of the three measured levels between the 2 groups.

Additional quantitative analysis of the caudal anastomosis for patients after a SCAR procedure (n = 6) showed a median SUV_max_ of 4.7 [3.8-5.5] and SUV_ratio_ of 2.2 [2.1-2.6] on the attenuation-corrected images. For patients in group 1, the median SUV_max_ was 4.2 [3.5-6.4] and the median SUV_ratio_ 2.3 [2.2-3.3]. For the 2 patients in group 2, the median SUV_max_ and SUV_ratio_ were 4.9 and 2.1, respectively. No significant difference in SUV_max_ or SUV_ratio_ was seen between the 2 groups (SUV_max_: *p* = .53, SUV_ratio_: *p* = .27). On the EARL reconstructed images, the median SUV_max_ was 3.6 [3.1-3.9] and the median SUV_ratio_ 1.8 [1.7-1.8] with no significant difference between the groups (SUV_max_: *p* = .27, SUV_ratio_: *p* = .80).

Quantitative analysis of the ^18^F-FDG uptake on the ascending aorta prosthesis could not be performed due to the diffuse uptake pattern in all cases.

## Discussion

The present study shows that in patients with ARAP, 18F-FDG uptake is present in the first year after surgery and has a homogeneous diffuse pattern and low to intermediate intensity on different areas of the prosthesis with no clear difference for any of the measured levels on both visual and quantitative analyses between the two groups (3 months vs 1 year after implantation) with exception of a small difference in the EARL SUVratio at the cranial anastomosis.

Since the inclusion of ^18^F-FDG PET/CT in the ESC guidelines of 2015, this imaging tool has become an important diagnostic method in suspected endocarditis in patients with a PHV. However, in patients with a concomitant ARAP which may be part of the infection process of IE or be solely infected, the value of ^18^F-FDG PET/CT is yet to be assessed. Misinterpretation of ^18^F-FDG PET/CT in patients with suspected infected ARAP can have severe therapeutic and prognostic consequences. Physiological ^18^F-FDG uptake due to normal healing response after surgery could be confused with pathological uptake or vice versa; pathological ^18^F-FDG uptake due to infection could falsely be interpreted as physiological uptake after recent surgery. Therefore, normal ^18^F-FDG uptake around the ascending prosthesis that is due to normal healing response after surgery, and its level of presence over the course of time after surgery, needs to be clarified in order to differentiate between physiological and pathological uptake intensity and pattern. One of the potential ways to differentiate between physiological and pathological ^18^F-FDG uptake is to treat patients with antibiotics if there is suspicion of pathological uptake and to repeat the PET/CT after 6 weeks. If the ^18^F-FDG uptake has not changed under antibiotic treatment, the uptake is probably false positive and in case of reduction of uptake intensity and form, the uptake is most certainly true positive. However, this way of differentiation is based on common sense and needs to be determined by studies and/or clinical trials and although logical, this approach is not preferred in clinical practice because of the side effects and costs that come along with antibiotic treatment. Follow-up studies should be based in an adequate interpretation, standardization, and reproducibility of the images and not in therapeutic response.

The usefulness of ^18^F-FDG PET/CT in suspected ARAP is described in the literature; however, this is limited to small case series and case reports.^11-13^ Lucinian et al. demonstrated in a retrospective series of 68 PET/CT’s made for suspected aortic root infection that heterogeneous uptake pattern with a high target-to-background ratio is associated with infection compared to non-infected aortic roots that had a more homogeneous uptake pattern with a relatively lower target-to-background ratio.^13^ However, caution in interpretation of this data is needed for determination of normal ^18^F-FDG uptake since all the included patients had suspicion of infection. In our study, we included only patients with no suspicion of infection and so demonstrating only physiological ^18^F-FDG patterns and intensity.

Roque et al. and our previous work recently demonstrated the normal ^18^F-FDG uptake patterns and intensity around prosthetic heart valves in the first year after prosthetic valve implantation in patients who did not undergo associated aortic surgery.^6,7^ Both studies found that ^18^F-FDG uptake shortly after valve implantation is relatively low and does not differ from the uptake 1 year after the implantation. Compared to the results of our current study, this is similar to the ^18^F-FDG uptake for the cranial anastomosis of the ascending aorta prosthesis and around the PHV. Very recently, a new study presented the first attempt to provide normal ^18^F-FDG uptake patterns on ascending aortic prosthetic grafts in the first year after implantation.^14^ This study corresponds in some ways with our study; however, there are some differences. First, some patients had undergone different types of surgery compared to our study (e.g., David procedure, supracoronary graft without PHV implantation, and reoperation); second, the post-operative PET/CT scans were made on different time points after surgery; and finally, the ^18^F-FDG uptake was measured for the total prosthesis only and not separately for the graft anastomoses. Their results showed a slight decrease in ^18^F-FDG uptake in the first year after surgery with no distinctive ^18^F-FDG uptake pattern which can be linked to non-infected prostheses.

Quantitative analyses on different locations of the ARAP showed only one small difference (EARL SUVratio of the cranial anastomosis) that was statistically significant and lower in the group 2 compared to group 1. Although other quantitative analyses of the cranial anastomosis showed no significant difference between the groups, the p-values were close to .05 and may become significantly different if the sample size was larger. This could mean that the 18F-FDG uptake on the cranial anastomosis could decrease over time. However, this conclusion cannot be made based on the results of this study. Whether the ^18^F-FDG uptake would decrease over a longer period of time than the first year is yet to be clarified with further research. However, theoretically, if there was no use of surgical adhesives, the inflammation process after surgery should decrease over time which can lead to decrease in ^18^F-FDG uptake.

Our study has some limitations, with the most obvious being the small sample size of the study. However, since little is known about normal 18F-FDG uptake around ARAP and the importance of normal reference values for correct interpretation of 18F-FDG PET-CT in suspected endocarditis, we feel the results provide valuable and clinically relevant information. A limitation of this study was that the scan was performed once in every patient and not multiple times in the same patient to actually see a change over time in the uptake patterns and SUV values. This approach was not deemed feasible due to the high radiation dose of multiple PET/CT scans in individual healthy patients. Excluding obese patients and patients with diabetes mellitus could also be seen as a limitation to the applicability of our results. Both conditions can affect the healing process following surgery and could therefore potentially impact ^18^F-FDG uptake. However, in order to prevent inadequate glucose levels prior to the PET and restrict the radiation exposure to patients, the exclusion of these patients was necessary. Furthermore, surgical adhesives such as Bioglue were not used in any patient, and this may impact the applicability of the results. Although all of the included patients underwent a preparatory low-carbohydrate diet for reducing myocardial uptake, 4 of the patients still had “high diffuse” ^18^F-FDG uptake of the myocardium and in total, 45% of the patients did not have fully suppressed myocardium and this could be seen as a potential confounder to the qualitative and quantitative ^18^F-FDG measurements, especially around the PHV.

In conclusion, after ARAP, 18F-FDG uptake seems to remain present in the first year after surgery with a low to intermediate intensity and mostly homogeneous diffuse patterns. There is no clear difference between patients scanned 3 months and 1 year after surgery. The use of 18F-FDG PET-CT in the first year after ascending aorta prosthesis implantation for the detection of infection of such prosthesis needs to be done carefully taking the normal variability into account to avoid mistakes. More studies are required in order to clarify the utility of PET/CT in the diagnosis of ARAP infection.

## New Knowledge Gained

Our study provides, as one of the first prospective studies, the normal ^18^F-FDG uptake of the ascending aortic prosthesis in the first year after implantation. These findings may help clinicians in the interpretation of ^18^F-FDG PET-CT in patients with suspected infection of the ascending aorta prosthesis.

## Supplementary Information

Below is the link to the electronic supplementary material.

Supplementary file1 (PPTX 152 kb)

Supplementary file2 (MP3 3614 kb)

## Abbreviations

^18^F-FDG: ^18^F-Fluorodeoxyglucose
ARAP: Aortic root and/or ascending aorta prosthesis
CT: Computed Tomography
EARL: European Association of Nuclear Medicine Research Ltd.
ESC: European Society of Cardiology
IE: Infective endocarditis
PET: Positron Emission Tomography
PVE: Prosthetic valve endocarditis
QVSH: Qualification Visual Score for Hypermetabolism
SCAR: Supracoronary ascending aorta prosthetic replacement
SUV: Standardized uptake value
TEE: Transesophageal echocardiography
TTE: Transthoracic echocardiography
