# Supplementary material for: Normal imaging findings after ascending aorta prosthesis implantation on 18F-Fluorodeoxyglucose Positron Emission Tomography with computed tomography
Source: J Nucl Cardiol. 2021 Oct 27;29(6):2938–48. doi: 10.1007/s12350-021-02826-0 (PMC9834100; doi:10.1007/s12350-021-02826-0)
Supplement: Supplementary file 1 — Supplementary file1 (PPTX 152 kb) [file 12350_2021_2826_MOESM1_ESM.pptx]

## Slide 1
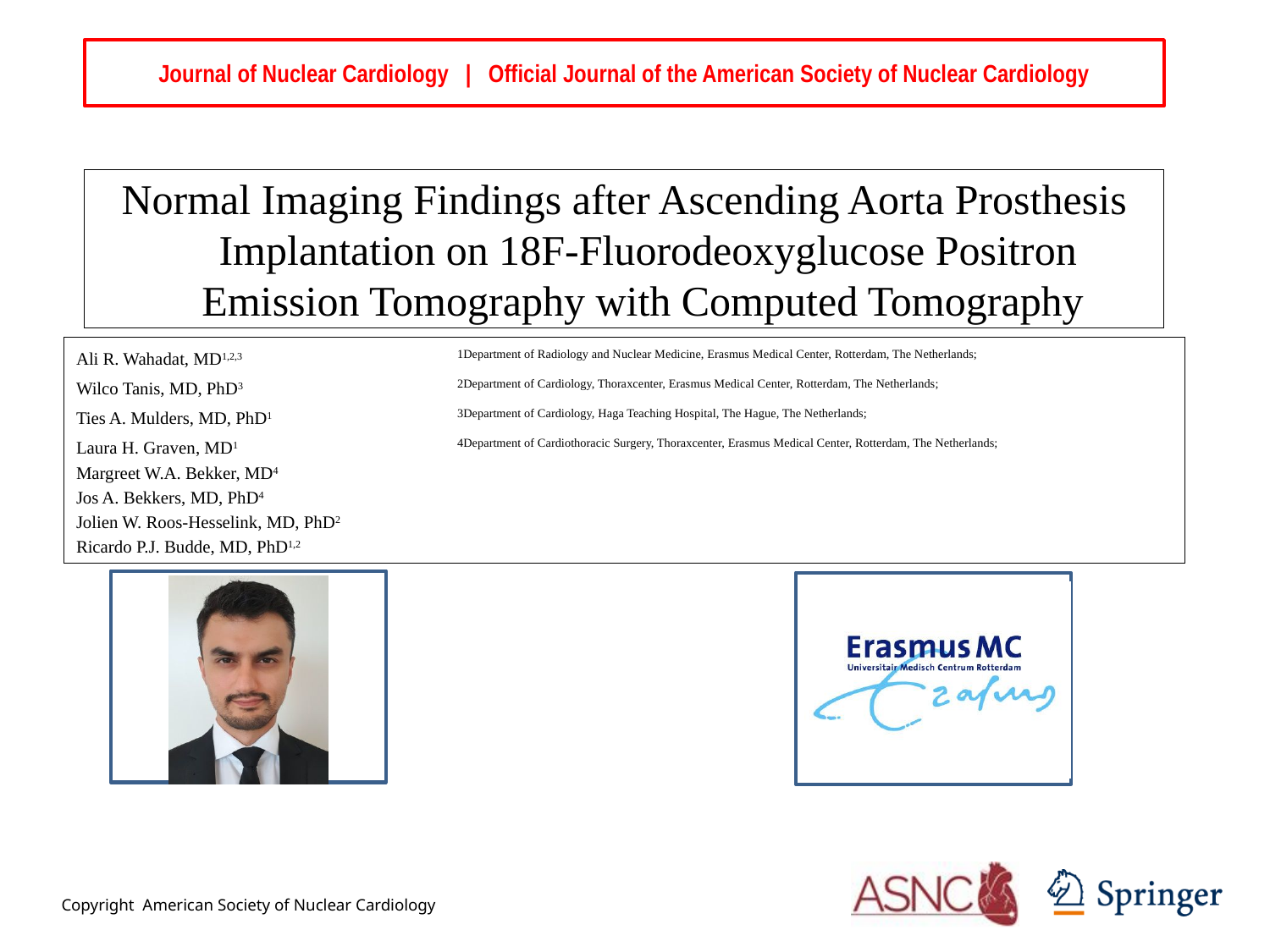

Journal of Nuclear Cardiology | Official Journal of the American Society of Nuclear Cardiology
# Normal Imaging Findings after Ascending Aorta Prosthesis Implantation on 18F-Fluorodeoxyglucose Positron Emission Tomography with Computed Tomography
Ali R. Wahadat, MD1,2,3 		1Department of Radiology and Nuclear Medicine, Erasmus Medical Center, Rotterdam, The Netherlands;
Wilco Tanis, MD, PhD3		2Department of Cardiology, Thoraxcenter, Erasmus Medical Center, Rotterdam, The Netherlands;
Ties A. Mulders, MD, PhD1		3Department of Cardiology, Haga Teaching Hospital, The Hague, The Netherlands;
Laura H. Graven, MD1		4Department of Cardiothoracic Surgery, Thoraxcenter, Erasmus Medical Center, Rotterdam, The Netherlands;
Margreet W.A. Bekker, MD4
Jos A. Bekkers, MD, PhD4
Jolien W. Roos-Hesselink, MD, PhD2
Ricardo P.J. Budde, MD, PhD1,2
Copyright American Society of Nuclear Cardiology

## Slide 2
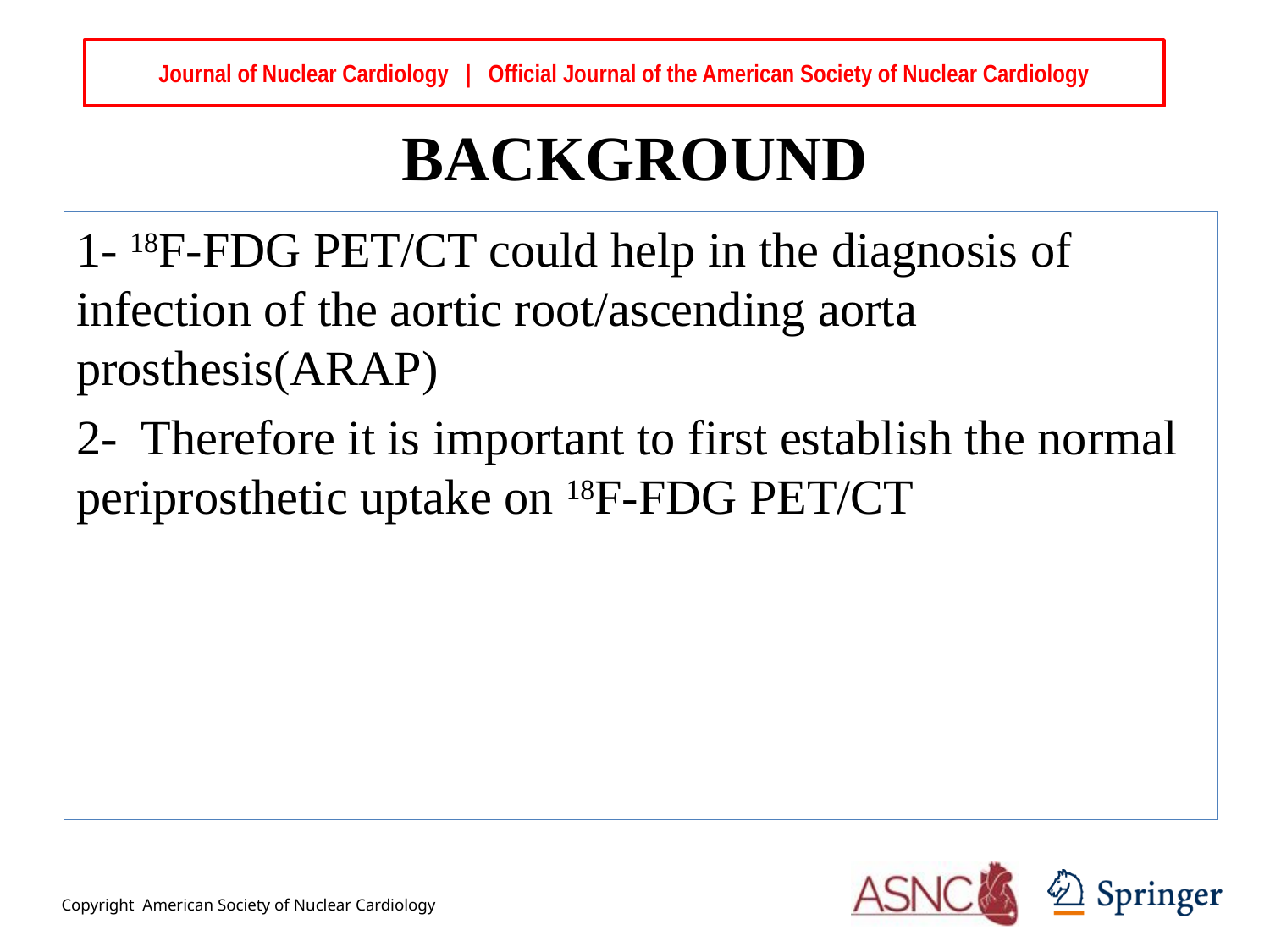

Journal of Nuclear Cardiology | Official Journal of the American Society of Nuclear Cardiology
# BACKGROUND
1- 18F-FDG PET/CT could help in the diagnosis of infection of the aortic root/ascending aorta prosthesis(ARAP)
2- Therefore it is important to first establish the normal periprosthetic uptake on 18F-FDG PET/CT
Copyright American Society of Nuclear Cardiology

## Slide 3
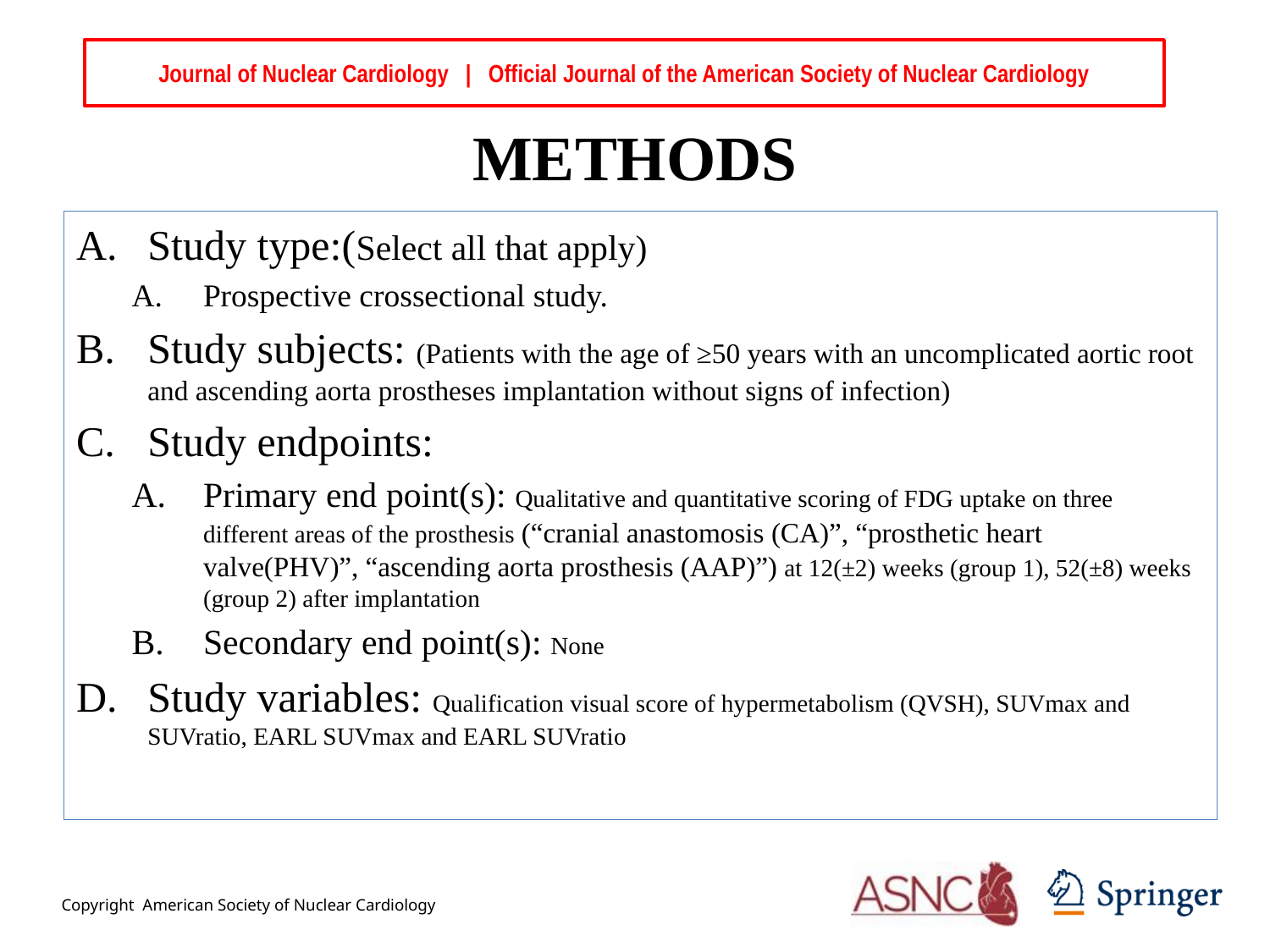

Journal of Nuclear Cardiology | Official Journal of the American Society of Nuclear Cardiology
# METHODS
Study type:(Select all that apply)
Prospective crossectional study.
Study subjects: (Patients with the age of ≥50 years with an uncomplicated aortic root and ascending aorta prostheses implantation without signs of infection)
Study endpoints:
Primary end point(s): Qualitative and quantitative scoring of FDG uptake on three different areas of the prosthesis (“cranial anastomosis (CA)”, “prosthetic heart valve(PHV)”, “ascending aorta prosthesis (AAP)”) at 12(±2) weeks (group 1), 52(±8) weeks (group 2) after implantation
Secondary end point(s): None
Study variables: Qualification visual score of hypermetabolism (QVSH), SUVmax and SUVratio, EARL SUVmax and EARL SUVratio
Copyright American Society of Nuclear Cardiology

## Slide 4
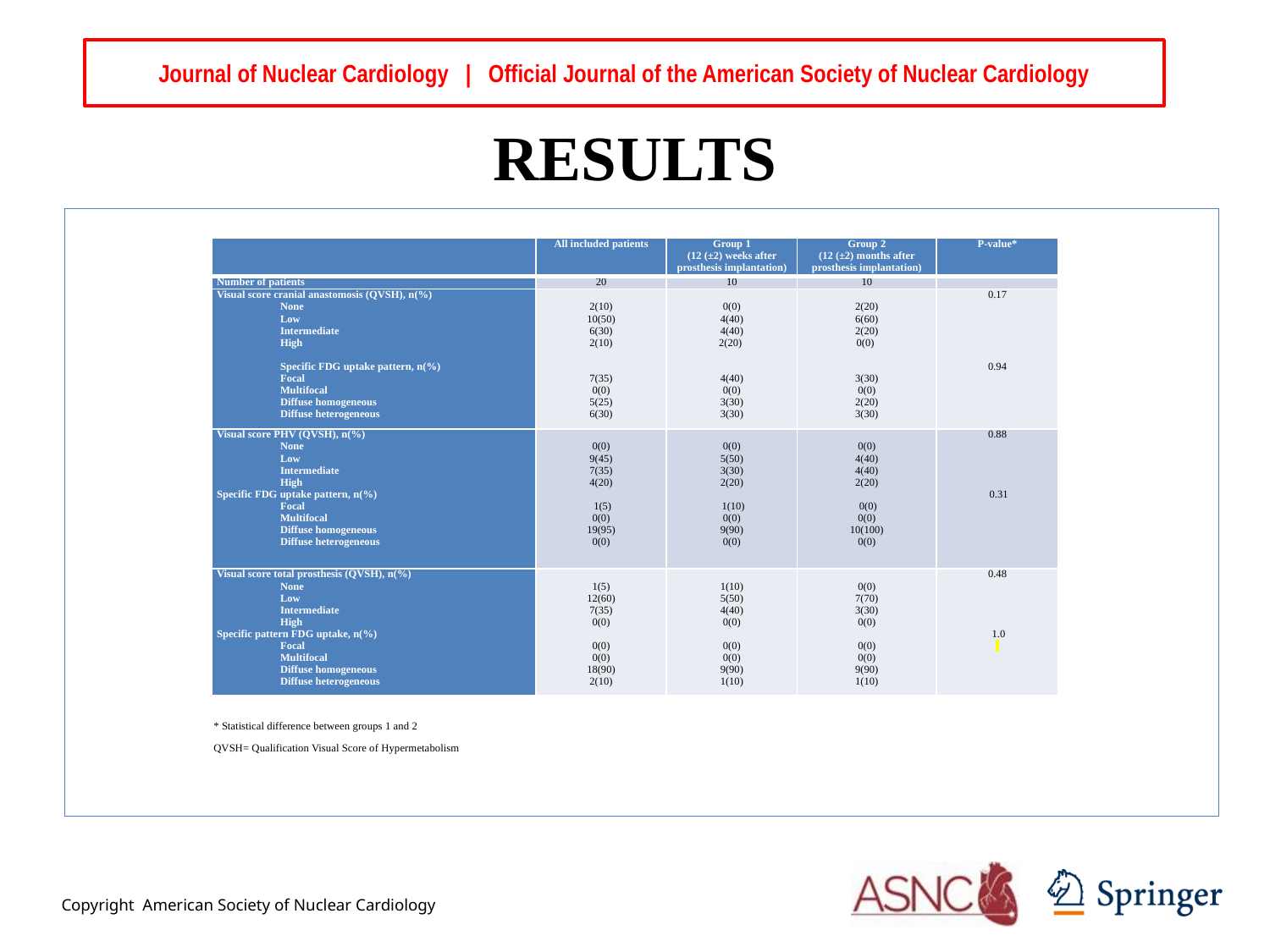

Journal of Nuclear Cardiology | Official Journal of the American Society of Nuclear Cardiology
# RESULTS
Insert a key table or a key figure
If figure, insert legend
| | All included patients | Group 1 (12 (±2) weeks after prosthesis implantation) | Group 2 (12 (±2) months after prosthesis implantation) | P-value\* |
| --- | --- | --- | --- | --- |
| Number of patients | 20 | 10 | 10 | |
| Visual score cranial anastomosis (QVSH), n(%) None Low Intermediate High Specific FDG uptake pattern, n(%) Focal Multifocal Diffuse homogeneous Diffuse heterogeneous | 2(10) 10(50) 6(30) 2(10)      7(35) 0(0) 5(25) 6(30) | 0(0) 4(40) 4(40) 2(20)    4(40) 0(0) 3(30) 3(30) | 2(20) 6(60) 2(20) 0(0)    3(30) 0(0) 2(20) 3(30) | 0.17         0.94 |
| Visual score PHV (QVSH), n(%) None Low Intermediate High Specific FDG uptake pattern, n(%) Focal Multifocal Diffuse homogeneous Diffuse heterogeneous | 0(0) 9(45) 7(35) 4(20)    1(5) 0(0) 19(95) 0(0) | 0(0) 5(50) 3(30) 2(20)    1(10) 0(0) 9(90) 0(0) | 0(0) 4(40) 4(40) 2(20)    0(0) 0(0) 10(100) 0(0) | 0.88          0.31 |
| Visual score total prosthesis (QVSH), n(%) None Low Intermediate High Specific pattern FDG uptake, n(%) Focal Multifocal Diffuse homogeneous Diffuse heterogeneous | 1(5) 12(60) 7(35) 0(0)   0(0) 0(0) 18(90) 2(10) | 1(10) 5(50) 4(40) 0(0)   0(0) 0(0) 9(90) 1(10) | 0(0) 7(70) 3(30) 0(0)   0(0) 0(0) 9(90) 1(10) | 0.48          1.0 |
* Statistical difference between groups 1 and 2
QVSH= Qualification Visual Score of Hypermetabolism
Copyright American Society of Nuclear Cardiology

## Slide 5
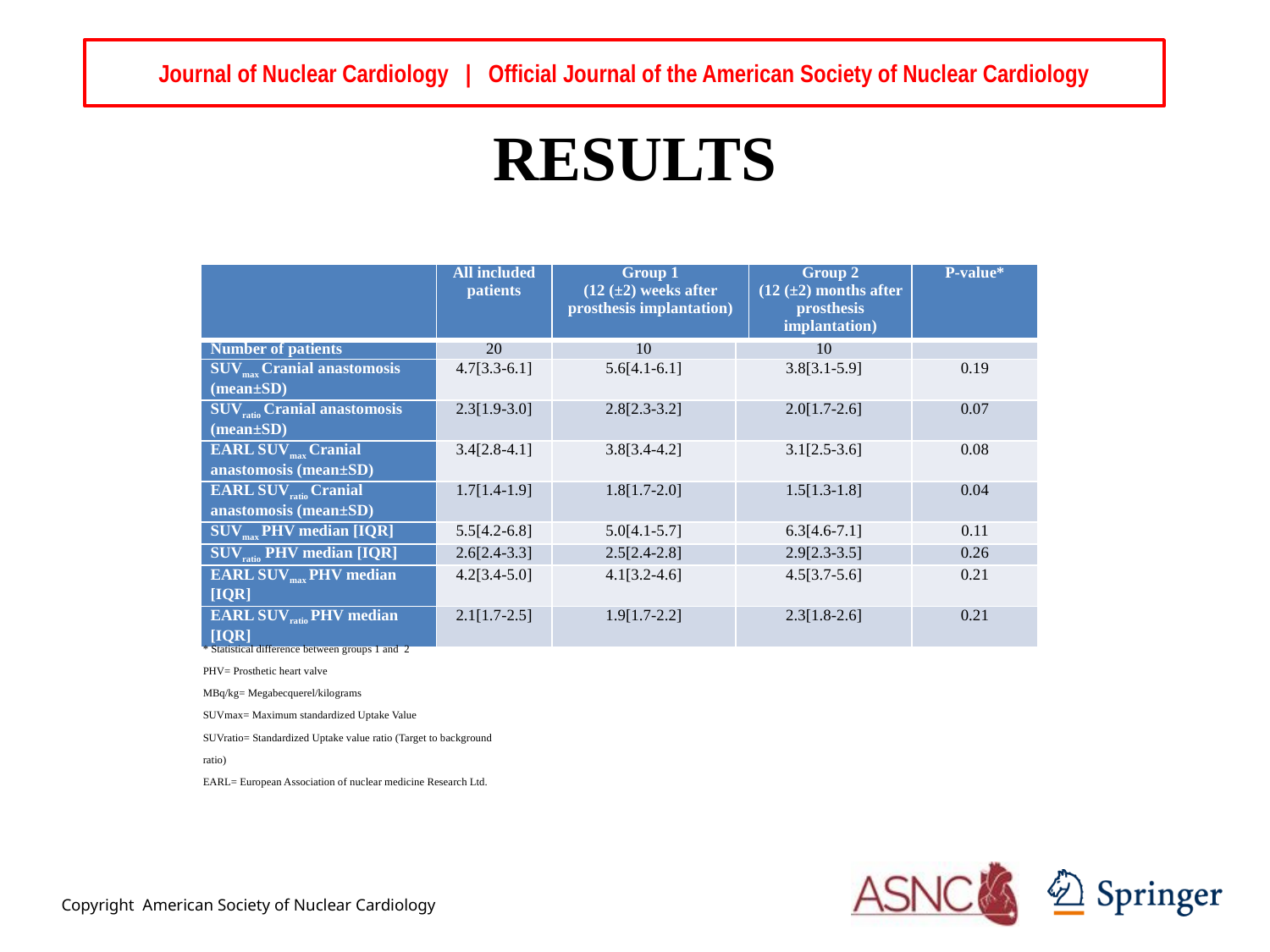

Journal of Nuclear Cardiology | Official Journal of the American Society of Nuclear Cardiology
# RESULTS
| | All included patients | Group 1 (12 (±2) weeks after prosthesis implantation) | | Group 2 (12 (±2) months after prosthesis implantation) | P-value\* |
| --- | --- | --- | --- | --- | --- |
| Number of patients | 20 | 10 | 10 | | |
| SUVmax Cranial anastomosis (mean±SD) | 4.7[3.3-6.1] | 5.6[4.1-6.1] | 3.8[3.1-5.9] | | 0.19 |
| SUVratio Cranial anastomosis (mean±SD) | 2.3[1.9-3.0] | 2.8[2.3-3.2] | 2.0[1.7-2.6] | | 0.07 |
| EARL SUVmax Cranial anastomosis (mean±SD) | 3.4[2.8-4.1] | 3.8[3.4-4.2] | 3.1[2.5-3.6] | | 0.08 |
| EARL SUVratio Cranial anastomosis (mean±SD) | 1.7[1.4-1.9] | 1.8[1.7-2.0] | 1.5[1.3-1.8] | | 0.04 |
| SUVmax PHV median [IQR] | 5.5[4.2-6.8] | 5.0[4.1-5.7] | 6.3[4.6-7.1] | | 0.11 |
| SUVratio PHV median [IQR] | 2.6[2.4-3.3] | 2.5[2.4-2.8] | 2.9[2.3-3.5] | | 0.26 |
| EARL SUVmax PHV median [IQR] | 4.2[3.4-5.0] | 4.1[3.2-4.6] | 4.5[3.7-5.6] | | 0.21 |
| EARL SUVratio PHV median [IQR] | 2.1[1.7-2.5] | 1.9[1.7-2.2] | 2.3[1.8-2.6] | | 0.21 |
* Statistical difference between groups 1 and 2
PHV= Prosthetic heart valve
MBq/kg= Megabecquerel/kilograms
SUVmax= Maximum standardized Uptake Value
SUVratio= Standardized Uptake value ratio (Target to background ratio)
EARL= European Association of nuclear medicine Research Ltd.
Copyright American Society of Nuclear Cardiology

## Slide 6
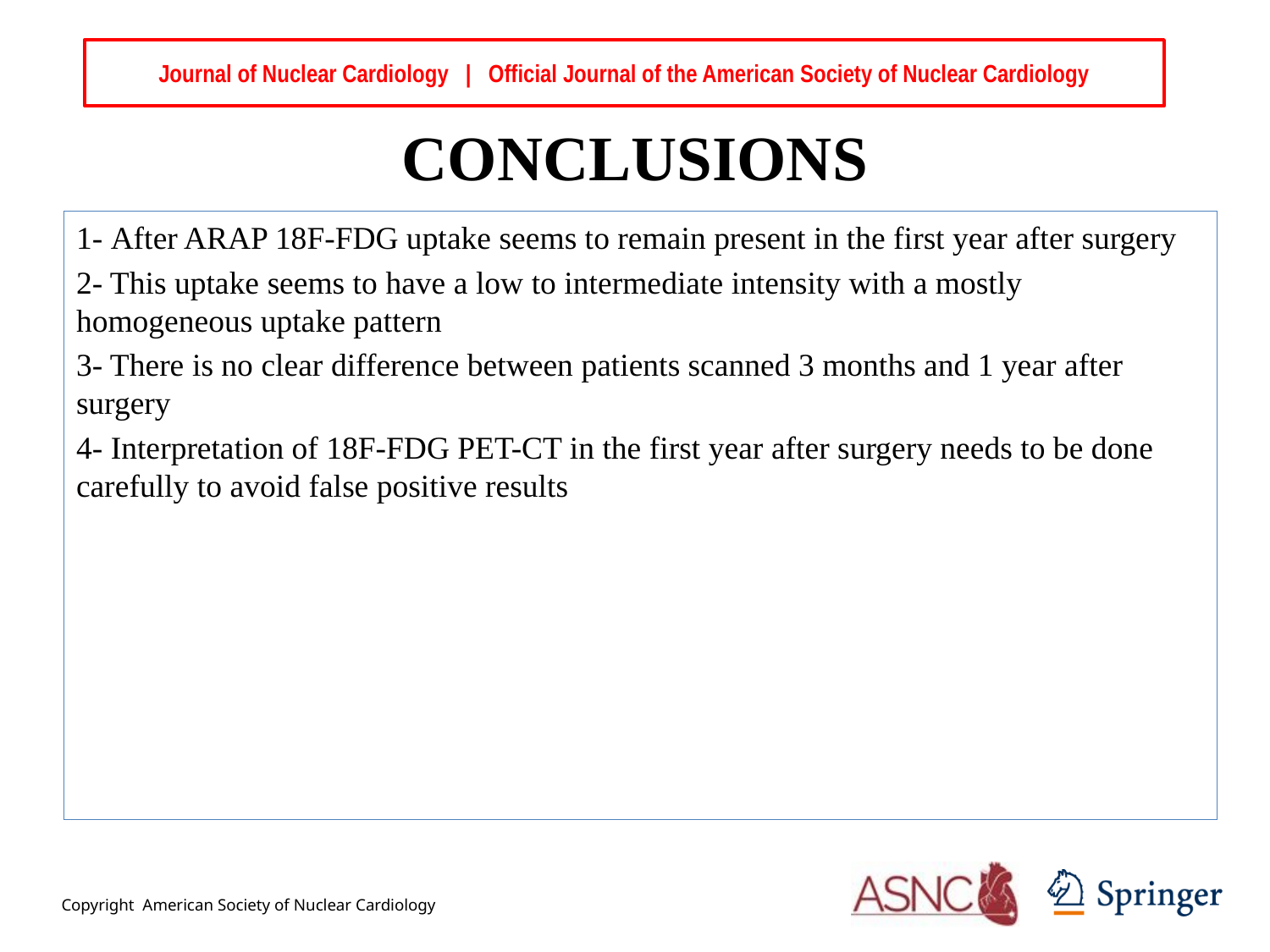

Journal of Nuclear Cardiology | Official Journal of the American Society of Nuclear Cardiology
# CONCLUSIONS
1- After ARAP 18F-FDG uptake seems to remain present in the first year after surgery
2- This uptake seems to have a low to intermediate intensity with a mostly homogeneous uptake pattern
3- There is no clear difference between patients scanned 3 months and 1 year after surgery
4- Interpretation of 18F-FDG PET-CT in the first year after surgery needs to be done carefully to avoid false positive results
Copyright American Society of Nuclear Cardiology
